# Unwanted Pregnancy and Associated Factors among Pregnant Married Women in Hosanna Town, Southern Ethiopia

**DOI:** 10.1371/journal.pone.0039074

**Published:** 2012-06-22

**Authors:** Belayneh Hamdela, Abebe G/mariam, Tizta Tilahun

**Affiliations:** 1 Department of Public Health Nursing, Hossana College of Health Sciences, Hossana, Ethiopia; 2 Department of Population and Family Health, Jimma University, Jimma, Ethiopia; Tehran University of Medical Sciences, Iran (Islamic Republic Of)

## Abstract

Of an estimated 210 million pregnancies that occur in the world each year, 38% are unplanned, out of which 22% end in abortion. In Ethiopia, the estimates of unintended pregnancy indicate that it is one of the major reproductive health problems with all its adverse outcomes. Women risk their lives in by seeking illegal abortions following unintended pregnancies. Thus, this study aims to determine the prevalence of unintended pregnancy and associated factors among pregnant married women residing in Hossana, Southern Ethiopia. A community-based cross-sectional study involving both qualitative and quantitative data collection methods was carried out in Hossana from April 02 to 15, 2011. 385 pregnant married women randomly selected from the census were included for the quantitative data and took in-depth interviews for the qualitative. Descriptive, binary and multiple logistic regression analyses were performed using SPSS version 16. Out of the total pregnancies, 131 (34%) were unintended and 254 (66%) were reported to be intended. A history of previous unintended pregnancy, the husband not wanting to limit family size, a desire for at least two children, the number of pregnancy 3–4 and parity of 5 and above were factors significantly associated with unintended pregnancy. With over one third of pregnancies unintended, having a previous unintended pregnancy, the number of previous pregnancies, and husbands’ disagreement over family size, and the desired number of children are factors that reproductive health programs should aim to focus on to reduce unintended pregnancy.

## Introduction

Unintended pregnancy is an important public health issue in developed and developing countries because of its negative association with the social and health outcomes for both mothers and children. Of the estimated 210 million pregnancies that occur throughout the world each year, about 38 percent are unplanned, out of which 22 percent end in abortion [1].

Ninety-five percent of unsafe abortions occur in developing countries. Millions more suffer long-term injuries from often life-threatening complications. In many poor countries, treatment of these complications consumes up to half of hospital budgets for obstetrics and gynecology [2].

It was estimated that current levels of unintended pregnancy, the prevalence of contraceptive use and the number of unintended pregnancies stem from early discontinuation and typical method failure rates. Every year in sub-Saharan Africa, approximately 14 million unintended pregnancies occur and a sizeable proportion is due to poor use of short-term hormonal methods [3].

A study from Nepal indicated that no single factor accounted for the high rates of unintended pregnancy; many factors contributed in this regard. Among them, this study has found that age of women, perceived ideal number of children, women’s age at first marriage, radio exposure, religion and knowledge of family planning methods are strong predictors of unintended pregnancy [4].

A Study finding confirms that a large proportion of Nigerian women are becoming pregnant when they do not want to. Among the factors a Nearly half said that their husband or non-marital partner did not want the pregnancy, one-third that they were too young or still in school, one-third that they were trying stop childbearing or space births and one-fifth that they had economic reasons. The results also imply that, unless unwanted pregnancies are prevented, many women will turn to abortion to avoid bearing children they are not prepared to have [5].

In southeast Ethiopia, a study in Harar town showed that from a total of 983 females aged 15–49 years who were interviewed, 225 (33.3%) reported that their most recent pregnancies were unintended [6].

Less is known about the factors associated with women’s unwanted pregnancies in Hossana town, southern Ethiopia. Thus, this study is aims to at determine the prevalence of unintended pregnancy and associated factors among pregnant married women residing in Hossana, southern Ethiopia.

## Methods and Materials

A community based cross-sectional study using both quantitative and qualitative methods was carried out in Hadiya zone, Hossana town from April 02–15, 2001. In Hossana there were eight kebeles (lowest administrative unit). Study from southern Ethiopia revealed that only 26.1% and 3.3% of the women received antenatal and delivery care services, respectively [7].

Census was conducted in all kebeles. The population for the quantitative survey included all pregnant married women residing in Hossana for at least six months prior to the survey. For the qualitative survey, health professionals working at reproductive health clinics (safe and post-abortion care health officers and nurses, family planning, antenatal care and urban health extension workers, clinical nurses) were included. The study populations included were purposely selected from the source population for an in-depth interview. For quantitative data, a sample from pregnant married women was considered.

To determine the sample size, a single population proportion formula using a prevalence of unintended pregnancy at 35% [6], a confidence level of 95%, and a 5% degree of precision, were used. A non-response rate of 10% was also used. From this, the final sample size calculated was 385. Multi-stage sampling technique was used.

A semi-structured and pre-tested questionnaire, guided by interviewer was used to collect the information. It was first prepared in English and then translated to Amharic and then translated back for consistency. Information collected included a questionnaire on demographic factors such as: maternal age; number of children; age at marriage and first sexual intercourse; previous unintended pregnancies; socio-economic and cultural factors such as education and ethnicity; husband desire for family size; desired number of children; experience of sexual violence; access to health services; and, questions on behavioral factors on modern contraceptive practice (including emergency contraceptives and breast feeding) and on reproductive history and unintended pregnancy. Four female college students and supervisor (a degree-holder) who knew the local language participated in data collection. Two days of training was given to data collectors and supervisors on the objectives of the study, the contents of the questionnaire, and particularly on issues related to the confidentiality of the responses and the rights of respondents. One week prior to data collection a pre-test was conducted in another Woreda (Ana Lemo) on 5% of the sample size.

Twelve in-depth interviews were conducted with 12 individuals using purposive and convenient techniques. Six health professionals participated in the study (health professionals working at reproductive health clinics: one from safe, one from post-abortion care service, one from family planning services, one from antenatal care and two urban health extension workers). In addition, clients attending antenatal care (four) and family planning (two) were interviewed.

Data collected were cleaned, edited, coded and entered to a computer, checked for missing values and outliers and analyzed using SPSS for windows version 16.0 (SPSS Inc. version 16.1., Chicago, Illinois). Simple descriptive frequency tables and then bivariate analyses were carried out. To identify the predictors of unintended pregnancy, a multivariable logistic regression model with unintended pregnancy as a dependent variable was constructed. Variables that showed significant association with unintended pregnancy on the bivariate analyses were entered into the adjusted logistic model. Interaction between variables was checked at the level of significance for the interaction term of P<0.05. The qualitative data were organized by selected themes, summarized manually and the results were triangulated with the quantitative findings.

Ethical approval was found from ethical review committee of Jimma University. Written consent was obtained from Hadiya zone health office and Hossana town health office administrations. Verbal informed consent was obtained from each respondent and confidentiality was assured before conducting the data collection, to respect the respondents participating in the study.

The following standard and operational definitions were used;

Unintended pregnancy is a pregnancy that is either mistimed (occurred earlier than desired) or unwanted (occurred when no children or no more children were desired) at the time of conception.

## Results

### Socio-demographic and Economic Characteristics

A total of 385 pregnant married women responded to the questionnaire. The majority of the respondents 329 (85.5%) were between 24–34 years of age. The mean age was found to be 27.6 (SD±4.79) years with a median of 28. Two hundred and fifteen (55.8%) of the study subjects had a family size of 1–4 with the mean as 4.56 (SD±2.19). Two-hundred and nineteen (56.9%) were protestants in religion and 245 (63.6%) were Hadiya in ethnic group (Table 1).

**Table 1:** Socio-Demographic Characteristics of Pregnant Married Women in Hossana Town, SNNP, April, 2011.

Variables	Frequency			Percentage	
**Age** n = 385					
15–19	8			2.1	
20–24	88			22.9	
25–29	154			40	
30–34	87			22.6	
35–49	48			12.4	
**Family size** n = 385					
1_4	215			55.8	
5_8	152			39.5	
9_12	18			4.7	
**Religion** n = 385					
Protestant	219			56.9	
Orthodox	108			28.1	
Muslim	27			7	
Catholic	25			6.5	
Others	6			1.5	
**Ethnicity** n = 385					
Hadiya	245			63.6	
Kambata	70			18.2	
Gurage	28			7.3	
Amhara	25			6.5	
Others	17			1.4	
**Education** n = 385					
Illiterate		43	11.2		
Read and write		21	5.5		
Elementary (1–8)		93	24.2		
High school (9–12)		134	34.8		
Higher education		94	24.4		
**Occupation** n = 385					
Housewife		167	43.4		
Government employee		89	23.1		
Self employee (vender)		58	14		
Student		35	9.1		
Farmer		17	3.9		
Others		25	6.6		

others: Jobless, self employee, etc.

others: Silte, tigre, oromo.

Other: Appopolistic religion.

Forty-three (11.2%) were illiterate, 93(24.2%) attended primary, and 94(24.4%) were in higher education. 63(48.1%) of those with unintended pregnancies and 165(65%) while of those with intended pregnancies had completed higher education. One hundred and sixty-seven (43.4%) were housewives, 89(23.1%) were government employees, 54(14%) were self-employed and 35(9.1%) were students. When asked about how they classified their family income as compared to their neighbor, 233(60.5%) classified themselves as average, 70(18.2%) well to do, 62(16.1%) poor, 12(3.1%) very poor and 8(2.1%) as rich (Table 1).

### Reproductive History

The mean ages at first sexual intercourse and at marriage were 19.86±2.76 and 20±2.78 respectively. One hundred and ninety-four (50.4%) of study subjects had first sexual intercourse in the age bracket of 20–24 years followed by the age group of 15–19 years, where there were 161(41.8%). One hundred and ninety-seven (51.17%) had married in the age group20–24; 158(41.03%) between 15–19 years, while others had married between 25–34 years 26(6.75%). The mean duration of marriage was 7.71±4.74. The mean age at first pregnancy was 20.6 (SD±2.8) with a median of 20 years. Two hundred and thirteen (55.3%) study participants had their first pregnancy in the 20–24 age bracket, followed by 127(33%) for the age group of 15–19 years (Table 2).

**Table 2:** Reproductive History of Pregnant Married Women in Hossana Town, SNNP, April 2011.

Variables	Frequency	Percentages
**Age at marriage** n = 385		
10–14	4	1.04
15–19	158	41.03
20–24	197	51.17
25–34	26	6.75
**Age at 1^st^ sexual intercourse** n = 385		
10–14	7	1.8
15–19	161	41.8
20–24	194	50.4
≥25	23	6.0
**Age at 1^st^ pregnancy** n = 385		
15–19	127	33.0
20–24	213	55.3
25–34	45	11.7
**Before current pregnancy** n = 385		
Ever pregnant	306	79.5
Never pregnant	79	20.5
**Number of pregnancy** n = 385		
1–2	145	37.7
3–4	115	29.9
≥5	125	32.5
**Previous unintended pregnancy** n = 385		
Yes	91	23.6
No	294	76.4
**Number of previous unintended pregnancy** n = 91		
1–2	86	94.5
≥3	5	5.5
**Previous birth gap** n = 385		
Less than three years	209	54.3
Greater than three years	98	25.5
No prior birth/1^st^ pregnancy	78	20.3
**Preferred gap b/n two consecutive pregnancy**		
Less than one year	3	0.8
One year	18	4.7
B/n one & two years	107	27.8
Greater than two years	257	66.8
**Method failure** n = 41		
Pills	24	58.5
Injectable	17	41.5
**Method taken according to instruction** n = 41		
Yes	11	26.8
No	25	61.0
Not instructed	5	12.2

Among respondents 34% constituted unintended pregnancy, 24.4% mistimed and 9.6% totally unwanted. For 79 (20.5%) of the study subjects were their first current pregnancy. As the number of times a women become pregnant increases, tendency to experience unintended pregnancy increases. A higher proportion of women with more than 5 pregnancies 58(46.4%) reported their pregnancy as unintended than women with 1–2 pregnancies 31(21.4%) (Table 3).

**Table 3:** Knowledge and Practice of Modern Contraceptives among Pregnant Married Women in Hossana Town, SNNP, April 2011.

Variables	Frequency	Percentage
**Knowledge**		
**Ever heard of MC** n = 385		
Yes	360	93.5
No	25	6.5
**Knowledge of MC methods** n = 385		
Knew at least one	91	23.6
Knew greater than one	248	64.4
**Advantage of MC** n = 385		
Knew non	44	11.4
Knew at least one	264	68.6
Knows greater than one	77	20.0
**Possible to prevent pregnancy after unprotected sex** n = 385		
Yes	171	44.4
No	81	21.0
I don’t know	133	34.5
**MC practice** n = 385		
Ever used	237	61.6
Never used	148	38.4

Among surveyed respondents, 91 (23.6%) of them had previously unintended pregnancies and 86 (94.5%) of these had had 1–2 previous unintended pregnancies. Previous unintended pregnancy was a risk factor for subsequent unintended pregnancy. A higher percentage of women with a history of previous unintended pregnancy had unintended pregnancy 53 (58.2%) as compared with women with no history of previous unintended pregnancy 78 (26.5%). Women with previous unintended pregnancy of 3 or more times were more likely to experience unintended pregnancy than women with 1–2 occasions of previous unintended pregnancy (Table 3). The most frequently reported reasons for failure to avoid unintended pregnancy were contraceptive failure 41 (31.3%) followed breast-feeding as contraception, 36 (27.5%). Other reasons were husband disapproval 18 (13.7%) and poor access to health services 5(3.8%).

Of those who reported contraceptive failure (n = 41), 24(58.5%) had used pills and 17 (41.5%) were injectable (Depo-Provera) users. Women who reported method failure as the reason were instructed but had not taken according to instruction; 11(26.8%) were instructed and had taken accordingly and 5 (12.2%) were totally uninstructed totally on specific methods used.

### Knowledge and Practice on Modern Contraceptives

The majority of the studied subjects 360(93.5%) had heard of modern contraceptives. Their numbers for each source of information included: 259 (67.3%) for health institutions, 137 (35.6%) for radio, 112 (29.1%) for television and 78 (20.3%) had heard from urban health extension workers. Two hundred and forty-eight (64.4%) knew more than one method of contraceptive. The numbers for the commonest sources of contraceptives were 185 (53.3%) for hospital and 118 (34%) for health centers. Two hundred and sixty-four (68.6%) knew at least one advantage of modern contraceptive methods and 77 (20%) knew more than one advantage, although 44 (11.4%) did not mention any advantage of modern contraceptives.

Two hundred and thirty-seven (61.6%) of study subjects had previously used modern contraceptive while 148(38.4%) had never used any methods of modern contraceptives. Injectable was the most frequently used method with 144(60.8%) followed by pills for 87(36.7%). Frequently reported reasons for non-use of modern contraceptives were: wanting to give birth 45(30.4%) and lack of awareness about modern contraceptive methods 40(27%). The mean age at first contraceptive use was 21.9 (SD±3.3). Most of them started at the age of 20–24 years 141(59.5%) and 14–19 years 50(21.1%) while others started at the age of 25–34 years 46(19.4%).

Binary and multiple logistic regression analysis was done using enter method to analyze factors associated with unintended pregnancy. On the binary logistic regression analysis, women’s age, previous unintended pregnancy, number of pregnancy, education, husbands’ desire for family size, lifetimes, and the desired number of children, were all associated with unintended pregnancy (Table 4).

**Table 4:** Association of Reproductive and Socio-Demographic Characteristics of Pregnant Married Women with Unintended Pregnancy, Hossana Town, SNNP, April 2011.

Variables	Unintended pregnancy		COR(95% CI)	AOR(95%CI)
	Yes Freq. (%)	No Freq. (%)		
**Previous unintended pregnancy**				
Yes	53(58.2)	38(41.8)	3.86(2.37,6.31)	2.76(1.55, 4.91)*
No	78(26.5)	216(73.5)	1	1
**Number of pregnancy**				
1–2	31(21.4)	114(78.6)	1	1
3–4	42(36.5)	73(63.5)	2.12(1.22, 3.67)	3.16(1.37, 7.3)*
≥5	58(46.4)	67(53.6)	3.18(1.87, 5.41)	5.6(1.62, 19.41)*
**Husband desire for family size**				
Agree	79(27.5)	208(72.5)	1	1
Disagree	43(57.3)	32(42.7)	3.54(2.09, 5.99)	3.24(1.69, 6.21)*
I don’t know	39.1)	14(60.9)	1.69(0.70, 4.07)	1.23(0.36, 4.17)
**Life time desired no of children**				
0–2	11(52)	10(47)	1.79(0.72, 4.42)	5.86(1.83,18.76)*
3–4	48(27)	127(72.6)	0.61(0.394,0.957	1.76(0.89, 3.48)
≥5	72(38.1)	117(61.9)	1	1
**Family size**				
1–4	59(27.4)	156(72.6)	1	1
5–8	60(39.5)	92(60.5)	1.72(1.11, 2.68)	0.79(0.34, 1.86
9–12	12(66.7)	6(33.3)	5.29(1.89, 14.74)	1.45(0.33, 6.38
**Mother education**				
None	23(53.5)	20(46.5)	3.01(1.55, 5.86)	1.0(0.44, 2.27)
Primary	45(39.5)	69(60.5)	1.71(1.06, 2.75)	1.07(0.61, 1.89)
Secondary and higher	63(27.6)	165(72.4)	1	1
**Mother age**				
<25 yrs	28(29.2)	68(70.8)	0.41(0.20, 0.84)	1.89(0.54, 6.59)
25–34	79(32.8)	162(67.2)	0.49(0.26, 0.91)	1.13(0.46, 2.76)
≥35	24(50)	24(50)	1	1

NB: * shows significant association at 95% ci.

For the multiple logistic regression analysis, having a previous unintended pregnancy, the number of pregnancies, husbands’ disagreement over family size, lifetime, and the desired number of children, were identified as factors for unintended pregnancy.

## Discussion

This study shows the magnitude of unintended pregnancy and factors associated with it, such as demographic, socioeconomic, socio-cultural, access to health information/services and knowledge and use of contraceptive methods on unintended pregnancy.

The prevalence of unintended pregnancy was found to be 34% (131). This finding is comparable with a similar study conducted Harar town in which the prevalence of unintended pregnancy was 33.3% and the national figure of 35% [6], [8]. On the other hand, the result is in contrast to the currently increasing awareness of modern contraceptive methods, availability of services and contraceptive prevalence rate. From this we recognize that having awareness and contraceptive by themselves did not avoid unintended pregnancy; appropriate counseling on methods and the proper intake of chosen methods is also necessary.

The most frequent reasons mentioned by the participants in this study for failure to avoid unintended pregnancy were contraceptive method failure, using breastfeeding, lack of awareness, and husband/partner disapproval. Husband disapproval was reported by less than one third (13.7%) which is analogous with a study conducted in Harar (11.6%) [6]. This is may be due to men’s desire for more children than women in both areas because of different socio-cultural contexts as mentioned above. Method failure in the current study was much higher than that of Harar (31.3% vs. 11.1%). On the other hand, lack of awareness in the present study was much lower than that of Harar [22.1% vs. 70.6%, 5]. This may be due to a timely increase in awareness and utilization of modern contraceptives [6].

The number of pregnancies was significantly associated with unintended pregnancy. Women with 5 or more pregnancies were 5.6 times more likely to report having unintended pregnancy as compared to women with 1–2 pregnancies [AOR = 5.6, 95% CI: 1.62, 19.41]. On the other hand, women with 3–4 pregnancies were 3.16 times more likely to report having unintended pregnancy than women with 1–2 pregnancies [AOR = 3.16, 95% CI: 1.37, 7.30]. This result was supported by the study conducted in Harar in which mothers with five or more pregnancies had a significantly increased likelihood of the pregnancy being unplanned [6].

Previous unintended pregnancy was shown to be a significant risk factor for current unintended pregnancy. Among surveyed respondents, 91 (23.6%) of them previously had unintended pregnancy and 86 (94.5%) of them had 1–2 previous unintended pregnancies while 5 (5.5%) had 3 or more previous unintended pregnancies. Taking those women who had no history of previous unintended pregnancy as a reference category to see the independent effect, women with previous unintended pregnancy at 2.76 times a higher risk of unintended pregnancy [AOR = 2.76, 95%CI: 1.55, 4.91].

Husband desire for limiting family size was shown to be associated with unintended pregnancy. Women whose husband disagreed to limit the number of family members were 3.24 times more likely to have unintended pregnancy than women whose husband agreed to limit the number [AOR+3.24, 95%CI: 1.69, 6.21]. This result is parallel to a study done in Indonesia which revealed that husbands’ desire for family size was significantly related to unintended pregnancy [9]. Women who disagreed with husbands about their family size were more likely to consider their pregnancy as unintended. This may be due to difference between women and men’s preferences for a number of children.

### Conclusion and Recommendation

Findings of this study indicate unintended pregnancy is one of the major reproductive health problems in the study area. Husband desire for family size, number of pregnancies a woman had, and ideal number of children a woman wanted were among significantly associated factors with unintended pregnancy. Thus, reproductive health programs, promoting husband and wife communication, family planning access and women empowerment should be done. Furthermore, it is recommended that attempts should be made to bring change in integrating basics and reducing mistimed and unwanted pregnancy by conducting further study.
